# Stigma from healthcare professionals and care-limiting behaviors in individuals with substance use disorders: a mixed-methods study

**DOI:** 10.1016/j.lanepe.2025.101587

**Published:** 2026-01-12

**Authors:** Mathias Luderer, Dorothea Stockreiter, Annette Binder, Laura Müller, Franca Burger, Nathalie Stüben, Andreas Reif

**Affiliations:** a Department of Psychiatry, Psychosomatic Medicine and Psychotherapy, Goethe University Frankfurt, University Hospital, Germany; b Department of General Psychiatry and Psychotherapy, Addiction Medicine and Addiction Research Section, University Hospital Tübingen, Tübingen, Germany; c DZPG (German Center for Mental Health), partner site Tübingen, Tübingen, Germany; d Department of Psychiatry and Psychotherapy, Ludwig-Maximilians-University Munich, University Hospital Munich, Nußbaumstraße 7, 80336, Munich, Germany; e Fraunhofer Institute for Translational Medicine and Pharmacology ITMP, Theodor-Stern-Kai 7, 60596, Frankfurt am Main, Germany

**Keywords:** Stigma, Substance use disorder, Healthcare avoidance, Healthcare professionals, Treatment barriers, Discrimination, Treatment discontinuation

## Abstract

**Background:**

Stigmatization of individuals with substance use disorders (SUDs) by healthcare professionals (HCPs) is a recognized problem, but its direct impact on patient treatment choices has not been systematically quantified. We aimed to provide first robust, quantitative metrics of non-disclosure, treatment avoidance, and treatment discontinuation for any medical treatment directly attributable to HCP stigma and to explore the lived experiences underpinning these behaviors.

**Methods:**

We conducted a prospective mixed-methods study with 119 adult inpatients with SUDs at a German university hospital (2021–2024). A self-developed questionnaire assessed stigma-related behaviors and their association with self-stigmatization. Qualitative data were analyzed using reflexive thematic analysis (RTA). A person with lived experiences contributed to writing up the manuscript.

**Findings:**

49.6% (95% CI 40.3–58.9; n = 59/119) reported non-disclosure of substance use, 36.1% (95% CI 27.5–45.5; n = 43/119) avoided necessary medical treatment, and 29.4% (95% CI 21.4–38.5; n = 35/119) discontinued treatment due to stigma. Internalized stigma significantly predicted all three outcomes (aORs 1.055–1.075, p ≤ .001). RTA identified “Institutional Stigma” (addiction as a “moral failing”), “Barriers to Care” (obstacles to respectful treatment), and “Cost of Disclosure” (negative consequences such as hostility after revealing substance use).

**Interpretation:**

Stigma from HCPs is a quantifiable contributor of treatment disengagement, representing a direct threat to patient safety and a major contributor to the SUD treatment gap. These findings underscore the urgent need for evidence-based interventions, including training HCPs across all specialties in non-stigmatizing communication, to improve healthcare engagement for this vulnerable population and narrow the substantial treatment gap.

**Funding:**

None.

**Research in context**

**Evidence before this study**

To situate our research and verify the existing evidence gap, we conducted a targeted literature search of PubMed and Google Scholar for articles published up to August 2021 (German and English articles only). Our search strategy included combinations of terms such as (“substance use disorder” OR addiction) AND (stigma OR discrimination) AND (“healthcare professional” OR doctor OR nurse). The goal was to identify studies that reported on patient-perceived stigma from healthcare professionals and its consequences for treatment engagement.

The search confirmed that negative attitudes from HCPs are a well-documented phenomenon. Existing qualitative studies consistently described patients' experiences of being judged or dismissed. However, our search revealed a critical gap: we found no studies that systematically quantified the prevalence of specific care-limiting behaviors—namely non-disclosure, treatment discontinuation, and healthcare avoidance—as a direct consequence of stigma in a healthcare setting. While this link was widely assumed, its quantitative impact remained unknown, providing a clear rationale for our study.

**Added value of this study**

This study provides the first robust quantification of non-disclosure, healthcare avoidance, and treatment discontinuation in the context of feared or experienced stigma from healthcare professionals. Our findings provide concrete figures showing that nearly half of the participants did not disclose their substance use, a third avoided necessary medical care, and over a quarter discontinued treatment for this reason. Furthermore, through mixed-methods analysis, this study connects these quantitative findings with rich qualitative data from patient narratives. This illuminates how patients experience stigma, identifying key themes such as perceiving addiction as a moral failing in the eyes of HCPs, having legitimate medical complaints dismissed as being “just (a symptom of) the addiction”, and facing direct hostility upon disclosure. This study also provides the first statistical evidence identifying internalized stigma as a powerful predictor for all three of these care-limiting behaviors, establishing a quantifiable link between individual self-stigmatization and treatment disengagement.

**Implications of all the available evidence**

Our findings confirm the previous assumption that stigma from HCPs towards individuals with SUD is a direct and substantial barrier to care with measurable, negative consequences on patient behaviour. These findings underscore an urgent need for policies and practices aimed at reducing stigma within all healthcare settings. The results imply that systemic interventions are required, focusing on educating HCPs about SUDs as a medical condition, providing training in non-stigmatizing verbal and non-verbal communication, and implementing harm-reduction strategies. Addressing the stigmatizing attitudes and behaviors of HCPs is crucial to breaking the vicious cycle where stigma leads to poor health outcomes, which in turn reinforces negative stereotypes. Future research should focus on developing and evaluating the effectiveness of these anti-stigma interventions in diverse clinical environments to improve healthcare access and outcomes for this vulnerable population.

## Introduction

Stigma can be defined as a “reaction that singles out individuals with certain characteristics (e.g., mental illness, substance use), devalues them”, and leads to stereotypes, prejudice, and discrimination. Substance use disorders (SUDs) are highly prevalent, cause a significant burden of disease, yet are subject to more severe stigma than other mental illnesses.^1^, ^2^, ^3^ Stigma has been identified as a major reason why most individuals with SUD still do not receive treatment for their condition at all.^4^ These stereotypes portray people with SUDs as “dangerous”, “unpredictable”, or as having a “bad character” and being responsible for their disease.^1^^,^^5^^,^^6^ The public stereotypes are internalized by the stigmatized individuals, resulting in self-stigma. This internalization may trigger the “why try effect,” as described by Corrigan et al.: drawing on social cognitive theory, their model explains that after internalizing public stereotypes (e.g., being morally flawed), some individuals experience diminished self-worth and reduced self-efficacy. Influenced by these negative self-beliefs, they may abandon aspirations such as seeking treatment, fostering hope for recovery, or pursuing other valued life goals (e.g., “why should I even try to change, if I am a worthless addicted person”).^7^, ^8^, ^9^ Ultimately, internalized and public stigma can lead to social exclusion and isolation, which in turn negatively impacts substance use.^1^^,^^10^ On a population level this impact of stigma on individuals with SUD has been examined. Less is known about how individuals with SUD perceive stigmatization from healthcare professionals (HCPs) and how these experiences affect their engagement with SUD and non-SUD healthcare.

Stereotypes against people with SUDs are particularly pronounced among healthcare professionals (HCPs). Helplessness, lack of knowledge and lack of cross-sectoral approaches lead to “self-perpetuating” stigma as the structural stigma in the healthcare system makes it “difficult for (HCPs) to facilitate successful treatment”, reinforcing stigmatizing attitudes.^6^

The need to reduce stigma is undisputed, both in the general population and in HCPs. Given the high prevalence of somatic illnesses among individuals with SUD, stigmatization by HCPs likely affects treatment for these conditions as well.^2^^,^^8^^,^^11^^,^^12^ Although it is widely accepted that stigma in the healthcare setting increases barriers to different kinds of treatment and increases risk of treatment discontinuation for individuals with SUD, this has, to our knowledge, not been quantitatively assessed. Also, the interplay between internalized stigma and stigmatizing experiences derived from HCPs and their association with treatment decisions has not yet been investigated.

To address this crucial knowledge gap, our study has two integrated aims. First, to quantitatively assess the prevalence of non-disclosure of substance use, healthcare avoidance, and treatment discontinuation resulting from stigma by healthcare professionals (HCPs), and to investigate whether these behaviors are predicted by internalized stigma. While previous qualitative studies have described such experiences, they often used smaller samples, limiting their generalizability, and a robust quantification of these care-limiting behaviors was missing.^7^^,^^13^^,^^14^ Second, we also qualitatively explored the lived experiences that drive these statistics.

## Methods

The study was conducted from September 2021 until August 2024 in a single center (Addiction Unit, Department of Psychiatry, Psychosomatic Medicine and Psychotherapy, University Medical Center Frankfurt am Main, Germany).

### Context

The unit provides comprehensive inpatient care for patients unable to achieve progress in outpatient settings. Treatment includes medically supervised detoxification and abstinence-oriented therapies (standard duration: three weeks; up to eight weeks for severe co-occurring disorders). A multidisciplinary team (physicians, psychologists, nurses, occupational therapists, and social workers) delivers integrated care addressing physical and mental health aspects. Admission is voluntary following physician referral or self-referral; court-ordered patients were excluded.

### Recruitment

Adult patients (18 years or older) with SUD (ICD-10 F1x.2; excluding tobacco) in inpatient treatment were included consecutively.

Exclusion criteria were acute psychotic or manic symptoms, acute suicidality, failure to provide informed consent, legal guardianship, or language barriers.

### Quantitative data

We developed a brief three-item questionnaire (Table 1) to assess non-disclosure, care avoidance, and treatment discontinuation due to stigma, as no existing validated instrument covered these outcomes. The items were designed for clarity and face validity based on clinical experience and existing qualitative literature. The items were rated on a four-point Likert scale from 0 to 3, with higher values indicating stronger agreement. The four-point Likert scale responses (e.g., ‘strongly agree’ and ‘agree’) were dichotomized into the binary outcome ‘agree’ vs. ‘disagree’. This approach was primarily chosen to enhance the clinical interpretability of the findings—whether or not a stigma-related behavior occurred—and to avoid a neutral midpoint. Furthermore, this binary distinction increases the statistical stability of the model given the sample size.

**Table 1:** Questionnaire to assess consequences of expected and/or experienced stigmatization by HCPs due to substance use (disorder).

1. For a long time, I didn't dare to disclose my substance use to medical staff because I was afraid to receive worse treatment then.
2. When I'm sick, I am reluctant to seek medical help, because I am afraid that the medical staff would treat me worse because of my addiction.
3. I have once discontinued a treatment because I was afraid that the medical staff would treat me worse because of my addiction.
4. Please describe an event, when you were treated exceptionally bad by medical staff due to your addiction.

A fourth item asked for an example of exceptionally bad treatment by medical staff due to the patients’ addiction. This item was analyzed qualitatively as described in the section below.

In addition to the self-developed questionnaire, internalized stigma was assessed with a modified German version of the Internalized Stigma of Mental Illness Scale, a 29-item instrument that evaluates the subjective experience of stigma using a 4-point Likert scale. The terms “mentally ill/mental illness” were exchanged with “addiction/addicted” leading to the Internalized Stigma of Addiction Scale (ISAI).^15^ A global sum score and subscores (alienation, adoption of stereotypes, discrimination, social withdrawal) were calculated, with higher scores indicating greater internalized stigma.

Demographics, diagnoses and substance use were extracted from routine clinical data.

#### Statistical analysis

The three primary outcome items from the self-developed questionnaire (non-disclosure, healthcare avoidance, and treatment discontinuation) were dichotomized for the regression analysis. Proportions are reported with two-sided 95% confidence intervals (CIs). To identify predictors for these outcomes, a series of binary logistic regression analyses were conducted. To ensure statistical power and prevent overfitting, we pre-specified a focused regression model. This included internalized stigma (ISAI total score) as the primary predictor, age and sex as standard demographic covariates to control for potential confounding, and the number of SUD diagnoses (more than one vs. one). A separate regression model was run for each of the three dichotomized outcomes using these pre-specified variables. Results from the regression analyses are reported as Odds Ratios (OR) with their corresponding 95% confidence intervals (CIs). All statistical analyses were performed using SPSS (Version 29.0), and statistical significance was set at an alpha level of p < .05.

### Qualitative data

Qualitative data were derived from open-ended responses in which participants described specific instances of stigmatizing treatment. Handwritten answers were transcribed verbatim and anonymized. To ensure accuracy, transcriptions were cross-checked against the original questionnaires by a member of the research team. The resulting text corpus was then utilized for the reflexive thematic analysis (RTA), commencing with data familiarization. The analysis and coding of the qualitative data were conducted manually by the researchers without the use of specialized qualitative data analysis (QDA) software or AI/LLM technology. This approach was chosen to facilitate a deep and direct reflexive engagement with the textual data, which is consistent with the methodology of reflexive thematic analysis.

The qualitative data analysis was underpinned by an interpretivist research paradigm, reflecting our focus on participants' subjective experiences and interpretations. RTA was used as the analytical method because of its ability to capture different aspects which are interlinked in a complex manner.^16^ The analytical approach was initially inductive. During the analysis, the stigma model according to Corrigan, as depicted in the introduction, was included deductively in the analytical processes (particularly in phase 4 + 5).^9^ The meanings were interpreted on a semantic as well as on a latent level.

Our analysis was oriented on the six-phases of RTA.^16^ A summary of the analysis process is shown in Supplemental Table S1. We used Reflexive Thematic Analysis Reporting Guidelines (RTARG) to ensure quality in research and report.^17^ To enhance transparency and comparability, we have provided the Standards for Reporting Qualitative Research (SRQR) checklist for our study in the Supplementary Material.^18^

### Reflexivity

Reflexivity is an integral component of RTA.^16^ We engaged in ongoing critical reflexivity, acknowledging our potential influence on interpreting sensitive stigma experiences. The qualitative analysts (ML, DS, AB), with clinical and research backgrounds in psychiatry/addiction medicine from different university hospitals, actively discussed their assumptions regarding stigma prevalence and disclosure impact. Through reflexive meetings and workshop feedback, we critically evaluated how our perspectives shaped coding and theme development, consciously working to center participant narratives (e.g., focusing on emotional experiences, carefully applying Corrigan's model). This multi-faceted reflexive process aimed to bolster the credibility and trustworthiness of the analysis, consistent with our interpretivist framework and reflexive TA methodology.

### Role of the lived experience perspective

To contextualize the research findings with an authentic narrative, co-author Nathalie Stüben was involved during the interpretation phase of the project. After the primary quantitative and qualitative analyses were completed by the core research team, the key findings (both statistical results and qualitative themes) were presented to her. Her role was to provide an independent commentary on these findings, drawing from her personal perspective. This approach was chosen to enrich the data with a narrative layer and to validate that the scientific results resonated with the real-world experiences they aim to represent.

### Role of the funding source

There was no funding for this study.

### Ethics approval

Ethical approval was granted by the Ethics Committee of the Faculty of Medicine, Goethe University Frankfurt (No. 2021-261) in September 2021, and the study was conducted according to the declaration of Helsinki and Good Clinical Practice. Eligible patients were approached by study staff who explained study procedures. All participants provided oral and written informed consent.

## Results

In total, N = 119 patients with substance use disorders were included in the study. The demographic and clinical characteristics of the participants are summarized in Tables 2 and 3.

**Table 2:** Demographic and clinical characteristics (N = 119).

Age (mean; SD)	43.57	12.53
Female sex (N; % yes)	43	36.13%
Male sex (N; % yes)	76	63.87%

Participants with non-medical use of … (last 30 days)	n	%
Alcohol	91	76.47%
Alcohol (heavy drinking)	87	73.11%
Any drugs	68	57.14%
Cannabis	41	34.45%
Amphetamines	14	11.76%
Cocaine	32	26.89%
Opioids	11	9.24%
Hallucinogens	4	3.36%
Volatile solvents	3	2.52%
Sedatives	18	15.13%
GHB	2	1.68%
Intravenous drug use	3	2.25%

Days with … (last 30 days)	Mean	SD
Alcohol use	11.68	9.66
Alcohol use (heavy drinking)	10.52	9.66
Drug use	6.37	10.04

GHB: Gamma-Hydroxybutyric Acid.

**Table 3:** ICD-10 substance use disorder diagnoses (N = 119).

Substance use disorder due to use of …	Dependence (F1x.2)		Harmful use (F1x.1)	
	n	%	n	%
Alcohol (F10.x)	90	75.63%	2	1.68%
Opioids (F11.x)	10	8.40%	0	0%
Cannabis (F12.x)	21	17.65%	4	3.36%
Sedatives or hypnotics (F13.x)	16	13.45%	0	0%
Cocaine (F14.x)	19	15.97%	7	5.88%
Amphetamine/other stimulants (F15.x)	8	6.72%	2	1.68%
Volatile solvents (F18.x)	1	0.84%	0	0%
Multiple substances (F19.x)	9	7.56%	1	0.84%

Our sample consisted primarily of individuals with alcohol, cannabis, stimulant (cocaine, amphetamine or other) use disorder, and with sedative use disorder. Less than 10% had opioid use disorder and only N = 3 (2.25%) reported intravenous substance use in the last 30 days before inclusion.

### Results quantitative analysis

**Fig. 1:**
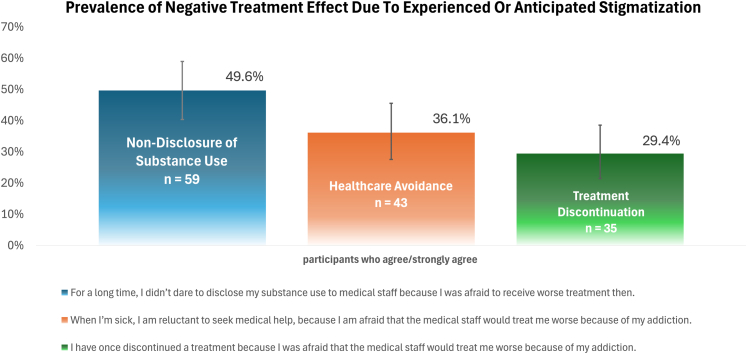
Prevalence of negative treatment effects (non-disclosure of substance use, avoidance of healthcare, treatment discontinuation) due to expected or experienced stigmatization by healthcare professionals (bars represent 95% confidence intervals).

All participants answered the first three items (Fig. 1): almost half of the participants (49.6% (95% CI 40.3–58.9; n = 59)) reported they have not disclosed their substance use from medical staff out of fear of worse treatment afterward. More than one third (36.1% (95% CI 27.5–45.5; n = 43)) reported avoiding medical treatment when they were sick, as they expected worse treatment due to their addiction. More than a quarter (29.4% (95% CI 21.4–38.5; n = 35)) reported having discontinued medical treatment due to the experience of being treated worse because of their addiction.

Internal consistency of the three items was acceptable (Cronbach's α = 0.756), suggesting they reliably measure the underlying construct of behavioral disengagement due to stigma.

The results of internalized stigma scale are presented in Table 4. To identify predictors for non-disclosure, treatment avoidance, and treatment discontinuation, multivariable logistic regression analyses were conducted, with results adjusted for age, sex, number of SUD diagnoses (more than one vs. one) and internalized stigma score (Table 5).

**Table 4:** Internalized stigma for addiction scale (ISAI).

	Mean	SD	25th percentile	Median	75th percentile
**Global scale**	53.46	14.35	41.0	54.0	64.7
**Subscales**					
Alienation	13.41	3.80	–	–	–
Adoption of stereotypes	16.54	4.80	–	–	–
Discrimination	11.22	3.33	–	–	–
Social withdrawal	12.37	3.97	–	–	–
Stigma resistance	11.58	2.50	–	–	–

SD: standard deviation.

**Table 5:** Multivariable logistic regression models predicting non-disclosure, treatment avoidance, and treatment discontinuation due to stigma.

Predictor	Non-disclosure of substance use	Treatment avoidance	Treatment discontinuation
	aOR (95% CI)	aOR (95% CI)	aOR (95% CI)
Internalized stigma	1.075 ∗∗ (1.037–1.109)	1.069 ∗∗ (1.034–1.105)	1.055 ∗∗ (1.021–1.091)
Age (per year)	0.973 (0.941–1.006)	1.004 (0.971–1.038)	0.989 (0.955–4.924)
More than one SUD diagnosis (excluding tobacco/nicotine)	0.424 (0.166–1.084)	1.004 (0.399–2.470)	0.518 (0.200–1.341)
Sex			
Female	Ref.	Ref.	Ref.
Male	0.898 (0.383–2.107)	0.683 (0.288–1.624)	1.959 (0.779–4.924)

aOR = Adjusted Odds Ratio; CI = Confidence Interval; Ref. = Reference category; aORs were adjusted for all variables listed in the table.

∗∗p ≤ 0.001.

Internalized stigma emerged as a highly significant predictor across all three outcomes (p ≤ .001). Each one-point increase in the internalized stigma score increased the odds of non-disclosure of substance use by 7.5% (Adjusted Odds Ratio [aOR] 1.075, 95% CI 1.037–1.109). Similarly, it increased the odds of treatment avoidance by 6.9% (aOR 1.069, 95% CI 1.034–1.105) and the odds of treatment discontinuation by 5.5% (aOR 1.055, 95% CI 1.021–1.091).

While these per-unit effects may appear modest, the cumulative impact across the observed range of ISAI scores is substantial. The interquartile range of ISAI scores in our sample spanned 23.7 points (25th percentile = 41.0; 75th percentile = 64.7; see Table 4). Comparing individuals at these percentiles, the odds ratios are 5.6 for non-disclosure, 4.9 for treatment avoidance, and 3.6 for treatment discontinuation—representing clinically meaningful differences.

In contrast, neither age nor sex were found to be statistically significant predictors for any of the outcomes in the adjusted models. Although younger age was associated with non-disclosure in the unadjusted bivariate analysis (Supplemental Table S5), this effect did not remain significant after controlling for internalized stigma and other covariates. The wide confidence intervals for sex and multiple SUD diagnoses reflect reduced statistical precision for these variables, likely attributable to the modest sample size and limited power to detect smaller effects for covariates beyond the primary predictor of interest.

Post-hoc power analysis (see Supplementary) for logistic regression (N = 119, α = 0.05, assuming R^2^ other X = 0.1) yielded achieved power of 0.42 for non-disclosure, 0.62 for treatment avoidance, and 0.72 for treatment discontinuation. While below optimal for some outcomes, internalized stigma remained a highly significant predictor across all three outcomes (p ≤ .001).

### Results qualitative analysis

38 participants (32%) provided free text examples of experienced stigmatization. The responses consisted of 1–184 words and were on average 30.39 words long.

Our analysis integrated semantic (explicit) and latent (implicit) content. At the semantic level, participants reported direct experiences, such as being blamed by HCPs (“It is your own fault,” ST-018) or denied care. The latent analysis moved beyond literal meaning to identify underlying assumptions and emotional implications. For example, quotes like “… typical for an alcoholic … obviously considered worthless” (ST-068) and feeling a nurse's “contempt and abysmal hate” (ST-025) highlight recurring concerns of being devalued and discriminated against. Through this dual-level, reflexive engagement with the data, we generated three overarching themes that capture these key patterns.

#### Theme 1- institutional stigma

Participants expressed that HCP did not take their SUD serious: either it was not depicted as a psychiatric illness, or the severity of it was downplayed [1.1, 1.2, 1.4]. Questioning the validity of SUD as a disease [1.1] was closely related to the assignment of guilt to the patient [1.2]. The belief that people are to blame for their condition was not limited to addiction alone. Patients described situations in which somatic conditions were attributed to their substance use and they were blamed for willingly causing these somatic consequences and increasing the workload of HCPs [1.2]. HCP were described as judging morally instead of acting professionally. This resulted in the experience of being reduced to a one-dimensional stereotype for example when the patient's treatment need was interpreted as an attempt to gain access to substances for ‘use’ [1.3] (Table 6).

**Table 6:** Theme 1–institutional stigma.

Theme 1	Brief overview
Institutional Stigma	Healthcare professionals often view addiction as a “moral failing” instead of a medical condition, leading to generalizations, oversimplification of the issue, and patients feeling dismissed or not taken seriously.
Quotes- theme 1	
1.1 [A trauma surgeon once played down the consequences of my addiction chummily, I was still young, I should simply keep my hands off it. It was well-intentioned, but I didn't feel like I was taken seriously. ST-149] 1.2 [“It is your own fault” from doctors and paramedics. Because of you, I have to get up early, because you don't pull yourself together. ST-018] 1.3 [No pain medication despite lumbago, but you have the possibility to get in the program to get the substance. ST-062] 1.4 [Just simply stop, fixation, typical for an alcoholic, can't become anything, must get deeper in the gutter, pigeonhole thinking] … [obviously considered worthless, even to be served, ungrateful, there would be better reasons to use money/time/medication. ST-068]	

In [1.4], the participant expressed a deeply negative, pessimistic and judging attitude towards patients with SUD resulting in a lack of dignity (“worthless”; “*there would be better reasons to use money/time/medication”).*

#### Theme 2–barriers to adequate care (“It's just the addiction”)

When HCPs knew about the patients’ SUDs, basics in medical and nursing care seemed to be disrupted. Patients reported that their concerns and needs were not taken seriously by HCPs, e.g., when questions regarding the medical procedures were denied [2.1] or their pain was ignored Table 7.

**Table 7:** Theme 2–barriers to adequate care (“It's just the addiction”).

Theme 2	Brief overview
Barriers to Adequate Care (“It's Just the Addiction”)	Patients perceive significant obstacles to receiving adequate, respectful care that maintains their dignity, often feeling their issues are solely attributed to their addiction.
Quotes- theme 2	
2.1 [A few years ago, I was often in hospital for alcohol detoxification. Every time I asked the senior physician some questions regarding my treatment or asked for something, consistently refused or said no. Other patients, who were with my on the ward but not for detoxification, had more success. ST-015] 2.2 [I went to the pulmonologist because of a pneumonia and flu and after he asked me if I was smoking (including cannabis) and I said yes he looked at me skeptically and blamed everything on it, even though I was really sick. ST-104] 2.3 [Perfect treatment in the first days, until the topic addiction came up. Afterwards I felt like the treatment was “canceled” and everything was blamed on consumption. ST-152] 2.4 [they just treat one once one is sobered out. Before that nobody pays any attention to you, not even if you have to go to the toilet. ST-026] 2.5 [For example, the ringing of the bell was ignored, because of my inability to move after the surgery I could no longer use the toilet by myself. They put me off, I wet the bed and was left lying in my feces for about 30 min. Afterwards, I got insulted how disgusting it was and because one had to wash me. ST-093]	

Others reported that the development of their somatic diseases or their symptoms were solely attributed to the patient's substance use [2.2; 2.3]. This also had an impact on treatment quality [2.3]. The barriers to receiving adequate care were described even more explicitly when treatment was terminated, denied or delayed, sometimes to an extent that basic human needs were ignored [2.4; 2.5].

#### Theme 3–cost of disclosure

This theme focuses on the direct negative consequences of seeking help as a person with SUD. Some patients decided to talk transparently with their HCPs about their SUD at some point [3.1], for some patients their SUD became known unwillingly [3.2]. The patients experienced a negative change of behavior of the HCPs. Many patients shared their experiences of being treated with contempt and rejection [3.1, 3.2, 3.3; 3.5], one even with open hostility (“abysmal hate”) [3.4]; (“angry (…) disappointed” [3.1]). This included non-verbal behavior (“even her facial expression (…) changed” [3.1]; “ignored me wherever they could or treated me roughly” [3.2]). The same applies to [3.3] where a participant reports “How they look at me and talk to me”. Although this quote does not explicitly state that the patient's SUD was exposed, stigmatizing behavior could only have occurred after labeling as a patient with SUD Table 8.

**Table 8:** Theme 3–cost of disclosure.

Theme 3	Brief overview
Cost of Disclosure	Revealing their substance use leads to direct negative consequences, including facing anger, disappointment, contempt, or hostility from healthcare staff.
Quotes- theme 3	
3.1 [In 2017 I received psychotherapy from [anonymized psychotherapist] in [anonymized city]. Our first communication took place by phone call. And at that occasion I didn't talk about alcohol consumption but rather about my problem with depression. At our first appointment I also told her about the alcohol consumption. She got very angry that I didn't mention it on the phone. Would she refuse to help me if I told her about alcoholism in advance? She asked me to create a chart in which I reported on which days I drink and how much. At the next appointment she recognized that I was drunk the day we had our first appointment (afterwards, not during the appointment). She got very angry and told me how disappointed she was. Even her facial expression and tone changed. I simply decided to be honest to get the help I need, and this is what I got back. ST-059] 3.2 [When I came to the hospital, the doctor asked me if I drink, which I denied, even though I was drunk. During the night I developed withdrawal symptoms, called the night doctor, confessed and let me give medication. Afterwards I got the impression the staff ignored me wherever they could or treated me roughly. ST-093] 3.3 [How they look at me and talk to me. ST-021] 3.4 [A few months ago. Emergency room [anonymized clinic]. Shortness of breath due to corona infection. Nurse probably knew that there was also an alcohol problem. Behavior of the nurse: Contempt and abysmal hate. Even the doctor on the ward told me later that it is known that the behavior of some nurses in the emergency room is indisposed and encouraged me to write a letter of complaint. ST-025] 3.5 [Once, it happened to me that I was treated ignorantly, unfriendly, inhumanly and incompetently in a hospital, psychiatric department, on the addiction ward. There were a few people of the nursing staff to whom this applies. But it definitely wasn't everyone, because it's never everyone. ST-147]	

Although the emergency room was often mentioned in the context of the cost of disclosure, other participants reported stigmatization on an addiction ward or in psychotherapy [3.1; 3.4]. Here, the last sentence “it definitely wasn't everyone, because it's never everyone” made clear, that the stigmatizing behavior of some HCPs on this ward is likely not representative for the whole team—however single actions seem to influence patients' choices.

## Discussion

Our findings demonstrate that stigma from HCPs has substantial, quantifiable consequences: nearly half of the participants reported non-disclosure of substance use, one third avoided necessary medical treatment, and more than a quarter discontinued treatment due to feared or experienced stigma.

Non-disclosure (49.6%) appears to serve as self-protection against anticipated devaluation and mistreatment. This rate aligns with existing literature, though the extent varies by setting and substance.^19^ Although the connection between anticipated stigma and non-disclosure seems intuitive, we found only one study that investigated this connection but did not quantify the extent and used a smaller sample of n = 33.^7^

When substance use cannot be addressed openly, treatment for SUD is delayed and efficacy of treatments for other conditions may be compromised.

With care avoidance reported by 36.1% and treatment discontinuation by 29.4%, our study quantifies for the first time the rate at which patients disengage from any medical treatment—not only SUD-specific care—due to stigma from HCPs. While previous research linked stigma to reduced SUD treatment initiation and continuation,^20^, ^21^, ^22^ our data reveal that stigma's impact extends across all healthcare domains. Participants frequently cited emergency departments as sites of particularly stigmatizing experiences, but also somatic treatment setting or psychotherapy.

Our mixed-methods approach illuminates how stigma translates into patient harm. The qualitative themes reveal the process: patients experience institutional stigma as their addiction is framed as moral failure; they encounter barriers to care when legitimate medical concerns are dismissed as “just the addiction”; and they face high costs of disclosure, including contempt and hostility after their SUD becomes known.

The strong, consistent association between the established ISAI scale and our novel behavioral items serves a dual purpose in our study. First, it provides crucial evidence for the construct validity of our self-developed questionnaire. That a validated measure of internal cognitive-emotional consequences of stigma significantly predicts concrete behavioral outcomes in healthcare settings demonstrates that our brief instrument, despite lacking formal validation, reliably captures stigma-driven disengagement. Second, it illuminates the mechanism underlying these behaviors: internalized stigma acts as the psychological bridge between external experiences of discrimination and patients' decisions to withdraw from care.

While the per-unit effect sizes appear small (aORs 1.055–1.075), this reflects the nature of a continuous predictor on a 29-item scale. The clinical impact becomes evident when considering the full range of observed stigma: individuals at the 75th percentile of internalized stigma (ISAI = 64.7) had 2.54-fold higher odds of non-disclosure, 2.31-fold higher odds of treatment avoidance, and 2.03-fold higher odds of treatment discontinuation compared to those at the 25th percentile (ISAI = 41.0). This substantial effect demonstrates that higher levels of internalized stigma translate into clinically significant behavioral disengagement from care.

Our study thus provides quantitative demonstration of the “why-try” effect in a healthcare context^9^: when patients internalize the message that they are devalued, their self-efficacy diminishes, leading them to abandon help-seeking not only for SUD but for any medical condition. This creates a vicious cycle where HCP stigma reinforces self-stigma, driving behaviors that may then confirm negative stereotypes among HCPs, further perpetuating institutional stigma.

Our findings provide crucial prevalence data from a European universal healthcare system. The 36% care avoidance rate in our sample is notably high—exceeding even the 29% found in a US study with much higher prevalence of intravenous drug use (78% vs. 2%).^23^ This is particularly alarming given Germany's minimal financial barriers to access.

In fragmented insurance-based systems like the US, disclosure costs may include insurance or employment consequences, potentially altering patients' calculations. That stigma remains such a powerful deterrent even in a universal system underscores that financial access alone is important but insufficient. Removing economic barriers must be coupled with dismantling interpersonal and systemic stigma. Our results strongly suggest that HCP stigma is a major, independent contributor to the substantial treatment gap in SUDs.

While our study was conducted in a single German hospital, the qualitative themes—institutional stigma, barriers to care, and cost of disclosure—echo findings from international studies,^24^ suggesting the fundamental mechanisms represent a global challenge requiring evidence-based solutions across diverse healthcare settings.

There is a conceptual proximity between our primary predictor, internalized stigma, and the stigma-related behavioral outcomes we measured. One could argue that it is expected for individuals with high internalized stigma to avoid healthcare. While we acknowledge this conceptual link, we interpret the consistent association not as a mere tautology, but as crucial evidence supporting the construct validity of our newly developed scale. The ISAI stems from a validated, 29-item scale assessing the internal cognitive and emotional consequences of stigma. In contrast, our three items assess specific behavioral consequences in health care. The finding that the internal experience of stigma predicts these concrete consequences provides a quantitative demonstration of the ‘why-try’ effect in the healthcare context and suggests that our brief questionnaire, while awaiting formal validation, effectively and reliably measures its intended constructs.

### Panel: view from a person with lived experiences

The background for my assessment of the results presented here is twofold. On the professional side, I have worked for many years on the topics of alcohol dependence, stigma, and recovery, both as an author and as a doctoral candidate. On the personal side, the issue is also part of my own history.

I belong to the large group of people who suffered from alcohol use disorder and never sought medical treatment for it. It didn't even occur to me for a long time. Sure, I drank a lot, crashes and injuries became more frequent — but I didn't drink every day, I didn't shake, still went to work, paid my taxes and looked fine. When all my drinking rules eventually failed, it dawned on me that I had a problem. In the end, I drank every few days until I blacked out, pouring most of my energy into hiding it. I knew I had to stop, but I was terrified of being ostracized and branded an “alcoholic.”

At the time, I didn't have a family doctor or a gynecologist. Only when I knocked out a front tooth while drunk or needed the morning-after pill, I went to a clinic. In these emergency situations, I didn't know how else to help myself. But seeing a doctor about my drinking? Unthinkable. I was too ashamed to admit what I saw as my failure. I already despised myself and couldn't bear the thought of rejection from anyone else.

So the findings presented here don't surprise me. I now see that internalized stigma as a ball and chain that kept me stuck. What if, back then at the clinic, I hadn't been met with a disparaging look, but with words like:

*“You’re not to blame. You’re not alone*. You can do this—and we can help you.” Maybe I could have been spared a few painful years.

Beyond this personal level, I believe changing the attitudes and behavior of healthcare professionals can positively impact society, helping to reduce the stigma surrounding people with SUD.

Nathalie Stüben

Stigmatizing language not only creates treatment barriers and leads to treatment discontinuation, it is also associated with diagnostic errors.^8^^,^^12^^,^^25^ The NIDA addressed this with their handout “words matter”. It provides guidance which expressions should be used preferably when talking with or about people with SUD. Stigma contributes to poorer health outcomes in individuals with SUD but also to the confirmation of prejudices, further perpetuating and reinforcing the stigma toward people with SUD among HCPs.^11^

We need to improve our skills in addressing the patients’ needs and providing them with guidance on their way to find help in a fragmented system, that also only has the capacity for the actual low treatment rates and is not built for those who are currently being deterred from treatment by stigmatization.

Several limitations should be considered. First, the questionnaire was not formally validated through psychometric testing. We developed this instrument due to lack of existing tools measuring these specific behavioral outcomes. While the acceptable internal consistency (α = 0.756) and convergent validity with internalized stigma support its utility, precise reliability and construct validity cannot be definitively established. This study provides an exploratory quantitative foundation upon which future research can develop validated instruments.

Second, post-hoc power analysis indicated suboptimal power (0.42–0.72), reflecting our modest sample size and small per-unit effects on a 29-item scale. However, the cumulative effect across the interquartile range represents clinically meaningful impacts (ORs 2.03–2.54), and achieving significance despite limited power suggests robust effects. Findings should be considered preliminary.

Third, the single-center design in a specialized inpatient unit limits generalizability. The sample consists exclusively of patients who successfully accessed inpatient care, potentially underrepresenting those with the most severe avoidance behaviors. However, this selection bias would likely lead to conservative estimates, as individuals most affected by stigma may never reach specialized inpatient treatment. That nearly half reported non-disclosure and over one-third avoided care despite being in treatment underscores the severity of the problem. Our qualitative themes align with international findings, suggesting the fundamental mechanisms are transferable across settings.^24^

Fourth, we relied on self-report from patients already in treatment, potentially underrepresenting barriers faced by those not seeking care.

Fifth, data collection by hospital staff posed social desirability risk, though interviews were conducted by researchers not involved in direct care. However, social desirability would suggest our high prevalence rates may be conservative estimates, with true extent potentially greater.

Sixth, questionnaire design choices (negative framing, four-point forced-choice scale, dichotomization) were deliberate but may have influenced responses and reduced granularity.

Seventh, we did not collect data on sexual orientation, SUD duration, or severity.

Finally, while our findings rely on subjective patient reports, this reflects the defining characteristic of stigma—not a methodological flaw. Patients' perceived reality dictates their actions. This underscores HCPs' responsibility to actively minimize any potential for patients to feel stigmatized, regardless of intent.

Stigma from HCPs contributes significantly to the treatment gap in SUDs. Our study provides the first robust quantification of behaviors linked to stigma, demonstrating high rates of non-disclosure of substance use, treatment avoidance, and treatment discontinuation in this context. We found that these behaviors are closely linked to patient experiences of institutional stigma, barriers to care, and the high personal cost of disclosure of substance use. To break the vicious circle where stigma perpetuates poor health outcomes and confirms stereotypes in HCPs, interventions must move beyond general education to specific, evidence-based training for HCPs across all specialties, including modules on communication and contact-based programs.^8^^,^^26^

Systemically, healthcare institutions must enforce non-stigmatizing language policies (see NIDA: “words matter”) and integrate harm reduction services to signal that patients with SUDs are worthy of compassionate, evidence-based care.^25^^,^^27^^,^^28^ This approach should also include training on non-verbal communication—addressing the very issue of ‘how they look at me’ that patients report—to ensure that respect is conveyed through both words and actions.

However, structural reform and training for HCPs are long-term goals. To bridge the gap immediately, the integration of individuals with lived experience into the treatment teams is a valid strategy. Peers can advocate for patients and help navigate a fragmented system but can also demonstrate recovery and reduce prejudices among HCPs through direct contact.

While conducted within a single German university hospital, the fundamental patient experiences uncovered echo findings from qualitative studies across the globe. While the specific prevalence rates of care disruption we quantified may vary slightly across different national contexts and healthcare systems, the fundamental mechanisms of stigma identified in our study likely represent a global challenge. The high rates of treatment discontinuation and healthcare avoidance we report represent a direct threat to patient safety and a significant contributor to the large treatment gap seen in individuals with SUDs. Understanding the interplay between institutional stigma, barriers to care, and the cost of disclosure is therefore of immediate importance to international clinicians, researchers, and policymakers seeking to develop evidence-based strategies to foster more effective and humane care.

Given the high rate of untreated individuals with SUD, the high impact of SUD on morbidity and mortality and the high burden of co-occurring psychiatric and somatic disorders in this population, we as healthcare professionals must admit: changing our stigmatizing attitudes and (verbal and non-verbal) behavior is more than overdue.^4^^,^^8^^,^^11^^,^^12^^,^^29^^,^^30^ As co-author Nathalie Stüben, a person with lived experiences, states: “Stigma feels like a second illness layered on top. If healthcare professionals took this into account, it could help ease a great deal of suffering.”

## Contributors

**CRediT**: Conceptualization: ML, DS; Data curation: ML, DS, AB; Formal Analysis: ML, DS, AB; Funding acquisition: AR; Investigation: ML, DS, LM, FB; Methodology: ML, DS; Project administration: ML, AR; Resources: AR; Software: ML, DS, LM, FB; Supervision: ML, AB; Validation: ML, DS, LM, FB, AB; Visualization: ML; Writing—original draft: ML; Writing—review & editing: ML, DS, AB, LM, FB, NS, AR.

## Data sharing statement

Data supporting this study cannot be made available as participants did not agree for their data to be shared.

## Declaration of interests

ML received a research grant from Oberberg Stiftung. He has received honoraria for lectures and presentations from Medice, Takeda, and Idorsia. He participated on advisory boards for Takeda and Recordati. He holds leadership roles as a board member of ICASA, board member of the German Association of Addiction Research and Addiction Therapy, and serves as Commissioner for Drugs and Addiction and Deputy Chairman of the Addiction Committee of the Hessian State Medical Association. He reports receiving author fees from Thieme and Elsevier.

AR reports grants or contracts from Medice and Janssen. He has received payment or honoraria for lectures, presentations, or educational events from Janssen, Boehringer Ingelheim, Compass, GH Research, SAGE/Biogen, LivaNova, Medice, Shire/Takeda, Newron, MSD, AbbVie, and Cyclerion. He participated on advisory boards for Takeda and Recordati. He holds leadership roles as President of the ECNP and Board member of the German Association for Psychiatry, Psychotherapy and Psychosomatics. He received author fees from Thieme.

AB reports funding from the Federal Institute for Public Health (project on alcohol consumption during pregnancy) and internal research funding of the Medical Faculty, University of Tübingen, Germany. She reports consulting fees for the review of the Master's program in Addiction Therapy at the Catholic University of Applied Sciences Cologne. She received honoraria for a CME article on the topic of alcohol from Thieme Publishing Group. She reports support for attending meetings from the German Center for Mental Health. She holds leadership roles as a Board member of the German Society for Addiction Research and Addiction Therapy (DG-Sucht) and as a German delegate to the UNODC Young Doctors Network of the United Nations.

NS reports being the author of the book “Frauen und Alkohol” (Women and Alcohol), published in December 2024. She reports paid speaking and/or reading from LE GOUVERNEMENT DU GRAND-DUCHÉ DE LUXEMBOURG, Ministère de la Santé et de la Sécurité sociale, pme Familienservice GmbH, GuS glass + safety GmbH, Fachklinik Altenkirchen, bus. | Bundesverband Suchthilfe e.V., DG Sucht, AWO Augsburg, Orvieto Academy, Her Career, AWO Traunstein, JETZT & MORGEN GbR, Gesundheitsamt Saarlouis, and Isar-Amper-Klinikum München. She declares other financial or non-financial interests as the founder and operator of the online platform “Ohne Alkohol mit Nathalie” (Without Alcohol with Nathalie), which offers two paid online programs in addition to several free, abstinence-oriented services.

All other authors declare no competing interests.

## Appendix A Supplementary data

Supplementary Figure and Tables

Translated Abstract
