# Supplementary material for: Stigma from healthcare professionals and care-limiting behaviors in individuals with substance use disorders: a mixed-methods study
Source: Lancet Reg Health Eur. 2026 Jan 12;63:101587. doi: 10.1016/j.lanepe.2025.101587 (PMC12828365; doi:10.1016/j.lanepe.2025.101587)
Supplement: Supplementary Figure and Tables [file mmc1.pdf]

SUPPLEMENTARY TABLE 1: PROCESS OF THE ANALYSIS OF QUALITATIVE DATA 2

SUPPLEMENTARY TABLE 2: FREQUENCIES PRIMARY OUTCOME (NON-DICHOTOMIZED VARIABLES), N = 119 3

SUPPLEMENTARY TABLE 3: FREQUENCIES PRIMARY OUTCOME (DICHOTOMIZED VARIABLES - STRONGLY AGREE VS. OTHER), N = 119 3

SUPPLEMENTARY TABLE 4: FREQUENCIES PRIMARY OUTCOME (DICHOTOMIZED VARIABLES - STRONGLY DISAGREE VS. OTHER), N = 119 3

SUPPLEMENTARY FIGURE 1: FREQUENCIES OF NON-DICHTOMOZIED PRIMARY OUTCOME VARIABLES (%, N = 119) 4

SUPPLEMENTARY TABLE 5: DEMOGRAPHIC AND CLINICAL CHARACTERISTICS BY OUTCOME STATUS........ 5

POST-HOC POWER ANALYSIS........ 6

SRQR CHECKLIST 7

Supplementary Table 1: process of the analysis of qualitative data

Phases of reflexive TA	data analysis procedure
1. Familiarization with the data, initial reflexive meeting	Reading and rereading the personal experiences. All responses were independently reviewed by 3 researchers before being discussed in a joint meeting (DS, AB, ML).
2. Creating initial codes	Following a reflexive team discussion, initial codes were developed to capture the most prominent shared meanings. Data segments were coded inductively, focusing on semantic and latent meanings (DS).
3. Developing initial themes	In an iterative process, the codes were related to each other and summarized in broader themes that contained similar patterns of meaning (DS, AB, ML).
4. Reviewing initial themes	The initial themes were refined by reviewing them against the created codes and the entire data set. Additionally, they were checked in meaningfulness by discussing them between the researchers as well as in a research workshop on qualitative methods. We adjusted the themes and focused more specifically on the perspective of those affected instead of our perspective as healthcare professionals. We identified three separate final themes.
5. Defining and naming the themes	After identification of the final themes, definitions describing their contents were created (DS, AB, ML).
6. Producing the report	Writing up of our results and linking them with the quantitative data; embedding the overall results into a wider scientific context. Attention was paid to both reflexivity and the link to the original data material (DS, AB, ML).

Supplementary Table 2: Frequencies Primary Outcome (non-dichotomized variables), N = 119

	Strongly agree	agree	disagree	Strongly disagree
**non-disclosure of substance use, n (%)**	25 (21)	34 (28·6)	18 (15·1)	42 (35·3)
**treatment avoidance, n (%)**	12 (10·1)	31 (26·1)	27 (22·7)	49 (41·2)
**treatment discontinuation, n (%)**	20 (16·8)	15 (12·6)	19 (16)	65 (54·6)

Supplementary Table 3: Frequencies Primary Outcome (dichotomized variables - strongly agree vs. other), N = 119

	Strongly agree	Agree, disargee, Strongly disagree
**non-disclosure of substance use, n (%)**	25 (21)	94 (79)
**treatment avoidance, n (%)**	12 (10·1)	107 (89·9)
**treatment discontinuation, n (%)**	20 (16·8)	99 (83·2)

Supplementary Table 4: Frequencies Primary Outcome (dichotomized variables - strongly disagree vs. other), N = 119

	Disagree, argee, Strongly agree	Strongly disagree
**non-disclosure of substance use, n (%)**	77 (64·7)	42 (35·3)
**treatment avoidance, n (%)**	70 (58·8)	49 (41·2)
**treatment discontinuation, n (%)**	54 (45·4)	65 (54·6)

Supplementary Figure 1: Frequencies of non-dichtomozied primary outcome variables (%, N = 119)

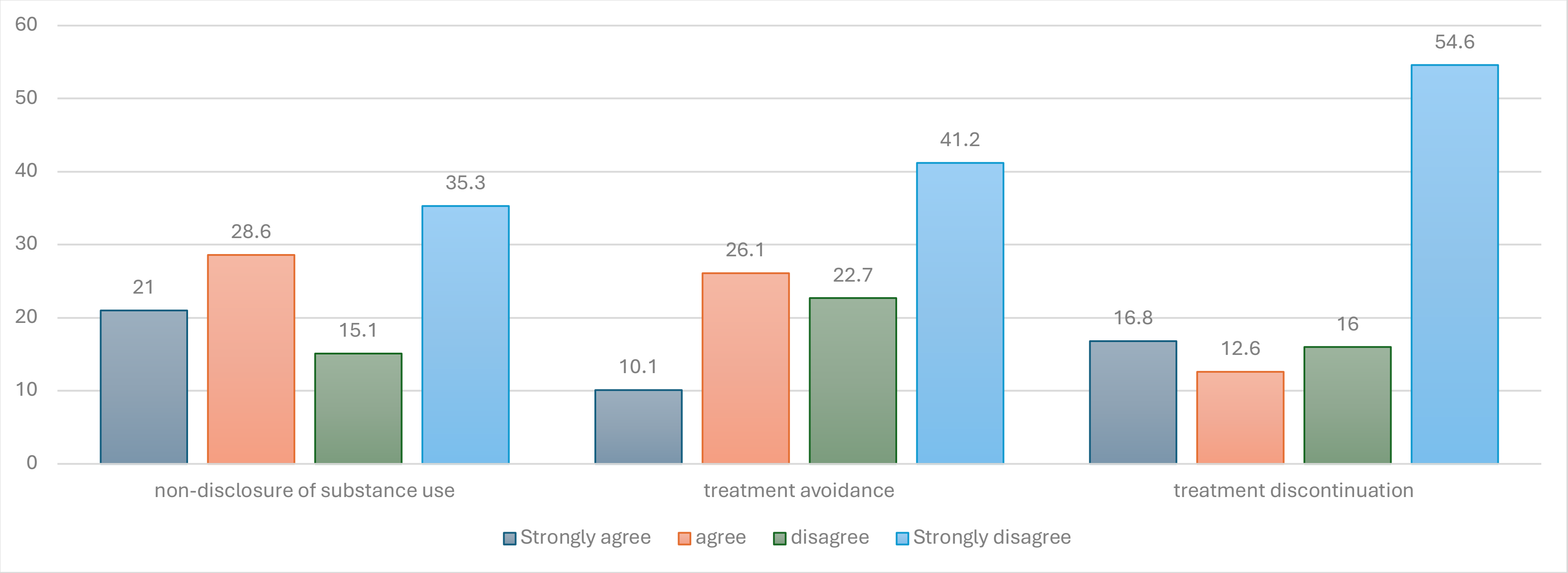

Supplementary Table 5: Demographic and clinical characteristics by outcome status

**Non-disclosure of substance use**					
	Yes		No		
	M, %	SD	M, %	SD	p
Age	41·19	12·223	45·92	12·480	·039†
Male Sex	N=36· 47·4%		N=40· 52·6%		·570‡
Female Sex	N=23· 53·5%		N=20· 46·5%		·570‡
ISAI global	59·30	13·038	47·71	13·308	< ·001†
Alienation	15·01	3·59	11·84	3·345	< ·001†
Adoption of stereotypes	18·22	4·337	14·85	4·684	< ·001†
Discrimination	12·364	3·048	10·10	3·240	< ·001†
Social withdrawal	13·73	3·759	11·04	3·72	< ·001†
Stigma resistance	12·21	2·305	10·96	2·541	·006†
**Treatment Avoidance**					
	Yes		No		
	M, %	SD	M, %	SD	p
Age	42·93	12·074	43·93	12·842	·676†
Male Sex	N=25· 32·9%		N=51· 67·1%		·472‡
Female Sex	N=18· 41·9%		N=25· 58·1%		·472‡
ISAI global	60·94	13·07	49·22	13·35	< ·001†
Alienation	15·63	3·467	12·16	3·408	< ·001†
Adoption of stereotypes	18·67	4·46	15·31	4·583	< ·001†
Discrimination	12·44	3·14	10·54	3·261	·002†
Social withdrawal	14·21	3·603	11·33	3·794	< ·001†
Stigma resistance	12·44	2·203	11·1	2·537	·005†
**Treatment discontinuation**					
	Yes		No		
	M, %	SD	M, %	SD	p
Age	41·63	11·280	44·38	12·990	·277†
Male Sex	N=25· 32·9%		N=51<br>67·1%		·301‡
Female Sex	N=10· 23·3%		N=33· 76·7%		·301‡
ISAI global	60·01	14·283	50·73	13·553	·001†
Alienation	14·89	3·793	12·79	3·656	·006†
Adoption of stereotypes	18·17	4·756	15·85	4·681	·016†
Discrimination	12·96	2·174	10·5	3·141	< ·001†
Social withdrawal	13·99	4·438	11·7	3·56	·004†
Stigma resistance	12·6	2·685	11·16	2·300	·004†

† Independent samples t-test; ‡ Chi-square test; M = Mean; SD = Standard Deviation; ISAI = Internalized Stigma of Addiction Index; SUD = Substance Use Disorder

Post-hoc power analysis

Post-hoc power analyses were conducted using G*Power 3.1.7 (Faul et al., 2009).

Faul F, Erdfelder E, Buchner A, Lang AG. Statistical power analyses using G*Power 3.1: Tests for correlation and regression analyses. Behav Res Methods 2009; 41: 1149-60.

Post-Hoc Power Analysis Non-Disclosure of Substance Use

z tests - Logistic regression
Options: Large sample z-Test, Demidenko (2007) with var corr
Analysis: Post hoc: Compute achieved power

Input:	Tail(s)	=	Two
	Odds ratio	=	1,075
	Pr(Y=1|X=1) H0	=	0,496
	α err prob	=	0,05
	Total sample size	=	119
	R^2 other X	=	0,1
	X distribution	=	Normal
	X parm μ	=	53,46
	X parm σ	=	14,35
Output:	Critical z	=	1,9599640
	Power (1-β err prob)	=	0,4181718

Post-Hoc Power Analysis Treatment Avoidance

z tests - Logistic regression
Options: Large sample z-Test, Demidenko (2007) with var corr
Analysis: Post hoc: Compute achieved power

Input:	Tail(s)	=	Two
	Odds ratio	=	1,069
	Pr(Y=1|X=1) H0	=	0,361
	α err prob	=	0,05
	Total sample size	=	119
	R^2 other X	=	0,1
	X distribution	=	Normal
	X parm μ	=	53,46
	X parm σ	=	14,35
Output:	Critical z	=	1,9599640
	Power (1-β err prob)	=	0,6161932

Post-Hoc Power Analysis Treatment Discontinuation

z tests - Logistic regression
Options: Large sample z-Test, Demidenko (2007) with var corr
Analysis: Post hoc: Compute achieved power

Input:	Tail(s)	=	Two
	Odds ratio	=	1,055
	Pr(Y=1|X=1) H0	=	0,294
	α err prob	=	0,05
	Total sample size	=	119
	R^2 other X	=	0,1
	X distribution	=	Normal
	X parm μ	=	53,46
	X parm σ	=	14,35
Output:	Critical z	=	1,9599640
	Power (1-β err prob)	=	0,7227311

Standards for Reporting Qualitative Research (SRQR)*

http://www.equator-network.org/reporting-guidelines/srqr/

Page/line no(s).

Title and abstract

**Title** - Concise description of the nature and topic of the study Identifying the study as qualitative or indicating the approach (e.g., ethnography, grounded theory) or data collection methods (e.g., interview, focus group) is recommended	p. 1 lines 135-141
**Abstract** - Summary of key elements of the study using the abstract format of the intended publication; typically includes background, purpose, methods, results, and conclusions	p. 1, lines 152– 221

Introduction

**Problem formulation** - Description and significance of the problem/phenomenon studied; review of relevant theory and empirical work; problem statement	p. 1-2, lines 257-274; lines 377-390
**Purpose or research question** - Purpose of the study and specific objectives or questions	p. 3, lines 495-497

Methods

**Qualitative approach and research paradigm** - Qualitative approach (e.g., ethnography, grounded theory, case study, phenomenology, narrative research) and guiding theory if appropriate; identifying the research paradigm (e.g., postpositivist, constructivist/ interpretivist) is also recommended; rationale**	p. 4, lines 670-672
**Researcher characteristics and reflexivity** - Researchers' characteristics that may influence the research, including personal attributes, qualifications/experience, relationship with participants, assumptions, and/or presuppositions; potential or actual interaction between researchers' characteristics and the research questions, approach, methods, results, and/or transferability	p. 4, lines 676-685
**Context** - Setting/site and salient contextual factors; rationale**	p. 3, lines 500-504
**Sampling strategy** - How and why research participants, documents, or events were selected; criteria for deciding when no further sampling was necessary (e.g., sampling saturation); rationale**	p. 3, lines 505-507
**Ethical issues pertaining to human subjects** - Documentation of approval by an appropriate ethics review board and participant consent, or explanation for lack thereof; other confidentiality and data security issues	p. 4, lines 688-690
**Data collection methods** - Types of data collected; details of data collection procedures including (as appropriate) start and stop dates of data collection and analysis, iterative process, triangulation of sources/methods, and modification of procedures in response to evolving study findings; rationale**	p. 3, lines 516-517; p. 4, lines 664-666

**Data collection instruments and technologies** - Description of instruments (e.g., interview guides, questionnaires) and devices (e.g., audio recorders) used for data collection; if/how the instrument(s) changed over the course of the study	p. 3, lines 509-517 p. 4, line 665
**Units of study** - Number and relevant characteristics of participants, documents, or events included in the study; level of participation (could be reported in results)	p. 4, lines 692-696 p. 6, Tables 2 & 3
**Data processing** - Methods for processing data prior to and during analysis, including transcription, data entry, data management and security, verification of data integrity, data coding, and anonymization/de-identification of excerpts	p. 4, lines 665-669
**Data analysis** - Process by which inferences, themes, etc., were identified and developed, including the researchers involved in data analysis; usually references a specific paradigm or approach; rationale**	p. 4, lines 667-675
**Techniques to enhance trustworthiness** - Techniques to enhance trustworthiness and credibility of data analysis (e.g., member checking, audit trail, triangulation); rationale**	p. 4, lines 676-685

Results/findings

**Synthesis and interpretation** - Main findings (e.g., interpretations, inferences, and themes); might include development of a theory or model, or integration with prior research or theory	p. 5, line 944 - p. 7
**Links to empirical data** - Evidence (e.g., quotes, field notes, text excerpts, photographs) to substantiate analytic findings	p. 5, lines 944-948 Tables 6, 7, 8 (on p. 7, 9, 10)

Discussion

**Integration with prior work, implications, transferability, and contribution(s) to the field -** Short summary of main findings; explanation of how findings and conclusions connect to, support, elaborate on, or challenge conclusions of earlier scholarship; discussion of scope of application/generalizability; identification of unique contribution(s) to scholarship in a discipline or field	p. 7, line 1247 - p. 9
**Limitations** - Trustworthiness and limitations of findings	p. 9, l. 1584-1592

Other

**Conflicts of interest** - Potential sources of influence or perceived influence on study conduct and conclusions; how these were managed	p. 11, lines 1748-1837
**Funding** - Sources of funding and other support; role of funders in data collection, interpretation, and reporting	p. 1, line 222 p. 4, lines 686-687

*The authors created the SRQR by searching the literature to identify guidelines, reporting standards, and critical appraisal criteria for qualitative research; reviewing the reference lists of retrieved sources; and contacting experts to gain feedback. The SRQR aims to improve the transparency of all aspects of qualitative research by providing clear standards for reporting qualitative research.

**The rationale should briefly discuss the justification for choosing that theory, approach, method, or technique rather than other options available, the assumptions and limitations implicit in those choices, and how those choices influence study conclusions and transferability. As appropriate, the rationale for several items might be discussed together.

Reference:

O'Brien BC, Harris IB, Beckman TJ, Reed DA, Cook DA. **Standards for reporting qualitative research: a synthesis of recommendations.** *Academic Medicine*, Vol. 89, No. 9 / Sept 2014
DOI: 10.1097/ACM.0000000000000388
