# Supplementary material for: Stigma from healthcare professionals and care-limiting behaviors in individuals with substance use disorders: a mixed-methods study
Source: Lancet Reg Health Eur. 2026 Jan 12;63:101587. doi: 10.1016/j.lanepe.2025.101587 (PMC12828365; doi:10.1016/j.lanepe.2025.101587)
Supplement: Translated Abstract [file mmc2.docx]

# Translated Abstract

**Hintergrund**

Stigmatisierung von Personen mit Substanzkonsumstörungen (SUD) durch medizinisches Fachpersonal ist ein bekanntes Problem. Der direkte Einfluss auf Entscheidungen für oder gegen Behandlungen wurde bislang jedoch nicht systematisch quantifiziert. Unser Ziel war es, erste belastbare quantitative Kennzahlen zu Nicht-Offenlegung von Substanzkonsum, Vermeidung und Abbruch jeglicher medizinischer Behandlung zu liefern, die direkt auf Stigmatisierung durch medizinisches Personal zurückzuführen sind. Außerdem sollten die diesen Verhaltensweisen zugrundeliegenden persönlichen Erfahrungen („Lived Experiences“) untersucht werden.

**Methoden**

Wir führten eine prospektive Mixed-Methods-Studie mit 119 erwachsenen stationären Patienten mit SUDs an einem deutschen Universitätsklinikum durch (2021 – 2024). Ein selbst entwickelter Fragebogen erfasste stigma-bezogene Verhaltensweisen und deren Assoziation mit Selbststigmatisierung. Die qualitativen Daten wurden mittels reflexiver Thematischer Analyse (RTA) ausgewertet. Eine betroffene Person wirkte an der Erstellung des Manuskripts mit.

**Ergebnisse**

49·6% (95% CI 40·3 – 58·9; n=59/119) berichteten über die Nicht-Offenlegung ihres Substanzkonsums, 36·1% (95% CI 27·5 – 45·5; n=43/119) vermieden notwendige medizinische Behandlungen und 29·4% (95% CI 21·4 – 38·5; n=35/119) brachen Behandlungen aufgrund von Stigmatisierung ab. Internalisierte Stigmatisierung war ein signifikanter Prädiktor für alle drei Ergebnisse (aORs 1·055 – 1·075, p ≤ ·001). Die RTA identifizierte „Institutionelles Stigma“ (SUD als „moralisches Versagen“), „Barrieren in der Versorgung“ (Hindernisse für eine respektvolle Behandlung) und den „Preis der Offenlegung“ (negative Konsequenzen wie Feindseligkeit nach Offenlegung des Substanzkonsums).

**Interpretation**

Stigmatisierung durch medizinisches Fachpersonal trägt quantifizierbar dazu bei, dass Behandlungen nicht begonnen oder abgebrochen werden. Dies stellt somit eine direkte Gefahr für die Patientensicherheit dar und ist ein wesentlicher Faktor für die Versorgungslücke bei SUDs. Diese Ergebnisse unterstreichen die dringende Notwendigkeit evidenzbasierter Interventionen (einschließlich der Schulung von medizinischem Personal aller Fachrichtungen in nicht-stigmatisierender Kommunikation), um die Inanspruchnahme der Gesundheitsversorgung für diese vulnerable Gruppe zu verbessern und die erhebliche Versorgungslücke zu verringern.

**Finanzierung**

Keine.
